# Excess Deaths and Immunoprotection during 1918–1920 Influenza Pandemic, Taiwan

**DOI:** 10.3201/eid1510.080811

**Published:** 2009-10

**Authors:** Ying-Hen Hsieh

**Affiliations:** China Medical University, Taichung, Taiwan

**Keywords:** Influenza, pandemic, Spanish flu, excess mortality, Taiwan, immunoprotection, viruses, dispatch

## Abstract

To determine the difference in age-specific immunoprotection during waves of influenza epidemics, we analyzed excess monthly death data for the 1918–1920 influenza pandemic in Taiwan. For persons 10–19 years of age, percentage of excess deaths was lowest in 1918 and significantly higher in 1920, perhaps indicating lack of immunoprotection from the first wave.

Recent studies have focused on quantifying the global effects of the influenza pandemic of 1918–1920 (*1*–*3*). This pandemic swept through Taiwan in 2 waves, at the end of 1918 and again in early 1920, causing devastating loss of human life. A report about the devastation brought by the first wave of the influenza epidemic, published in February 1920 (*4*), indicated that as of December 15, 1918, a total of 779,522 persons (20.8% of the population) had been infected and 25,394 persons had died from influenza; case-fatality rate was 3.26% (*4*,*5*). Although the number of infections decreased dramatically in early 1919, a second wave of the epidemic at the end of that year created another severe death toll.

Previous studies have shown that excess deaths, similar to those noted in the temperate zones, were also observed in Taiwan, which is in a tropical–subtropical zone, during periods of previously recognized influenza epidemics (*6*,*7*). A recent study has also shown Taiwan to be an evolutionarily leading region for global circulation of influenza virus A (H3N2) (*8*). Therefore, we analyzed the 1918–1920 pandemic in Taiwan to contribute to understanding of and preparation for possible future pandemic events.

## The Study

Using data from the 1895–1945 Statistical Abstract of Taiwan (*9*), we compared monthly deaths during the 2 waves of epidemics in 1918 and 1920 with deaths during corresponding nonpandemic periods of the adjacent years. For example, we compared monthly deaths for November and December 1918 with the mean deaths for November and December 1916–1917 and 1919–1922. Statistically significant excess deaths were computed by detecting the data points at which the all-cause deaths exceeded the mean of the adjacent years +2 SDs (*6*,*10*). Excess deaths, computed from the mean number of deaths at these data points, were then used to ascertain the effect of the pandemic on deaths during these periods. During 1918–1920, population data were divided into 3 major groups: Taiwanese (95.2%), Mainland Chinese (0.57%), and Japanese–Korean (4.2%). However, only records of all-cause deaths for Taiwanese and Japanese were available and used in our analysis.

**Figure 1:**
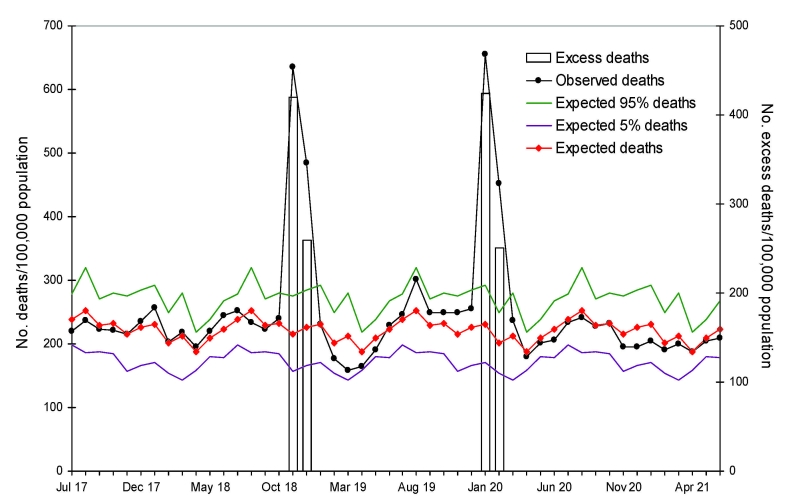
Monthly all-cause and excess death rates, Taiwan, July 1916 through June 1922. Monthly averages for excess deaths exclude those of the pandemic years 1918 and 1920. Bars indicate excess deaths per 100,000 population during the pandemic months of November–December 1918 and January–February 1920.

Figure 1 gives the mean monthly number of all-cause deaths and 95% confidence intervals (CIs) for each month during 1916–1922, excluding the known anomaly months (the 2 epidemic waves) of November–December 1918 and January–February 1920. The number of deaths increased markedly during the anomaly months. When we plotted the anomaly points against the actual number of deaths, we noted that the anomaly points were significantly >2 SDs above the means and that substantial excess deaths had indeed occurred. Moreover, we estimated the excess deaths during these 2 waves by subtracting the mean number of all-cause deaths from the number of deaths during the anomaly periods (Table 1). Because the 2 waves of epidemics had overlapped the adjacent months, we also included increases during these months.

**Table 1:** Excess all-cause deaths of Taiwanese and Japanese persons during 2 influenza epidemic waves in Taiwan, 1918–1920*

Time period	Observed no. deaths	Expected no. deaths†	SD	Excess no. deaths (95% CI)	Excess no. deaths, by wave‡ (95% CI)
First wave					
1918 Oct§	8,725	8,366	489	359 (0–1,337)¶	24,907 (21,426–29,008)
1918 Nov	23,156	8,042	768	15,114 (13,578–16,650)	
1918 Dec	17,658	8,224	793	9,434 (7,848–11,021)	
Second wave					
1919 Dec§	9,319	8,224	793	1,095 (0–2,682)¶	26,141 (20,572–32,845)
1920 Jan	23,906	8,478	973	15,429 (13,482–17,375)	
1920 Feb	16,466	7,466	955	9,001 (7,090–10,911)	
1920 Mar§	8,625	8,009	630	616 (0–1,877)¶	

*CI, confidence interval. †Expected monthly no. deaths was computed from mean no. deaths for that month during 1916–1922, excluding the epidemic month. Total excess no. deaths for these 2 periods = 51,048 (95% CI 41,998–61,853). ‡Including the excess no. deaths in adjacent months. §Excess no. deaths for these months are not statistically significant (i.e., not >2 SD above mean). ¶Max (0, lower bound).

**Figure 2:**
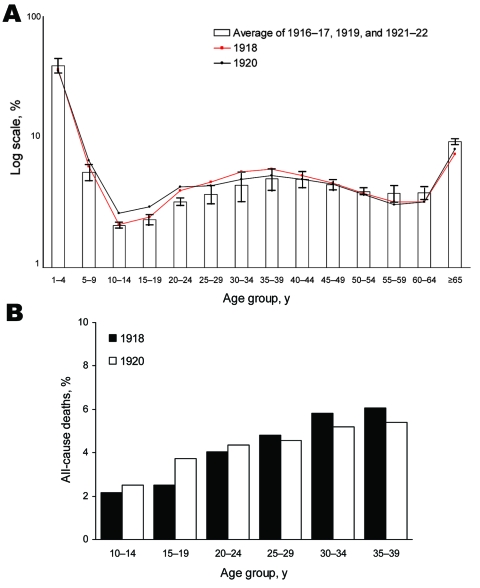
A) Percentages (in log scale) of all-cause deaths in Taiwan, by age group. Error bars indicate 95% confidence intervals. B) Percentages of all-cause deaths for persons 10–39 years of age in 1918, grouped by 5-year age groups.

We used age-specific data on deaths to quantify the effect of the 1918–1920 influenza pandemic on each age group. Because monthly age-specific death data were not available (*9*), we used yearly age-specific all-cause death data to quantify age-specific excess deaths during 1918 and 1920. Figure 2, panel A, gives the age-specific percentages of all-cause deaths of Taiwanese persons during 1917–1921 only. The percentages of all-cause deaths by age group were computed for 1918 and 1920 and compared with the respective averages of percentages for the adjacent years (1917, 1919, and 1921).

For persons 5–39 years of age, percentages of all-cause deaths for 1918 and 1920 were clearly higher than those for the average of adjacent years; for persons >55 years of age, they were lower. In addition, deaths were higher in 1918 than in 1920 for persons 25–39 years of age, but deaths were higher in 1920 for those 5–24 years of age. The excess percentages of deaths for 1918 and 1920 in age groups 10–19, 20–29, and 30–39 years were computed by subtracting the average percentages of deaths in these age groups during the adjacent years from the respective true percentages of deaths in these age groups during 1918 and 1920 (Table 2).

**Table 2:** All-cause deaths for Taiwanese persons during 1916–1922, by age group, and excess percentages of age-specific deaths for 1918 and 1920*

Year	Total no. deaths	Age group, y							
		10–19			20–29			30–39	
		No. (%) deaths	Excess % deaths (95% CI)		No. (%) deaths	Excess % deaths (95% CI)		No. (%) deaths	Excess % deaths (95% CI)
1918	124,677	5,836 (4.68)	0.19 (0–0.48)‡		11,028 (8.85)	1.88† (1.1–2.67)		14,804 (11.87)	2.78† (0.02–5.54)
1920	119,477	6,888 (5.77)	1.27† (0.09–0.56)		10,579 (8.85)	1.89† (1.11–2.68)		12,305 (10.30)	1.20 (0–3.96)‡

*CI, confidence interval. †The excess percentages for these age groups are statistically significant. ‡Max (0, lower bound).

The percentages of excess deaths were most significant for persons 20–39 years of age in 1918 and 10–29 years of age in 1920. When we compared the 1918 and 1920 waves, the percentage of excess deaths decreased during the second wave for persons 30–39 years of age, was almost identical for those 20–29 years of age, and was significantly higher for those 10–19 years of age. Even for those 5–9 years of age, the percentage of deaths was higher during 1920 than either during 1918 or for the average of adjacent years (Figure 2, panel A).

Our estimate of 1.38% (95% CI 1.14–1.68) excess deaths for Taiwan is close to the estimate of 1.44% (95% CI 1.40–1.48) by Murray et al. (*3*). However, their estimate was based on calculations of 3-year excess deaths for 1918–1920 over the preceding 3 (1915–1917) and the following 3 years (1921–1923). During 1919–1920, a cholera outbreak caused 2,693 deaths in 1919 and 1,675 deaths in 1920, which might have skewed their estimate of excess deaths for Taiwan. In contrast, our 1920 estimate, obtained by using higher resolution monthly data (more precise than yearly data), accounted for only the first 3 months of the year. Although the exact months of the cholera outbreak are unknown, we can reasonably assume that the excess deaths caused by cholera during these 3 months were substantially fewer than excess deaths from the entire cholera outbreak during 1919–20.

Ample literature describes the unusual age-specific death patterns for the 1918–1920 pandemic (*11*–*13*). To compare the age-specific differences between the 2 waves, we compared the percentages of all-cause deaths of persons 10–39 years of age in 1918 with the corresponding percentages of all-cause deaths for the same groups of persons, who 2 years later were 12–41 years of age (Figure 2, panel B).

Within the 10–39-year age group in 1918, the age groups with the lowest percentage of excess all-cause deaths in 1918 (10–19 years) had markedly increased deaths in 1920, and the age groups with a higher percentage of excess all-cause deaths in 1918 (30–39 years) had noticeably decreased deaths in 1920 (Table 2; Figure 2, panel B). This finding could be explained in part by acquired immunoprotection by those age groups during the first wave, thus giving credence to the belief that the 2 waves were caused by the same virus strain.

## Conclusions

In 1918, the epidemic swept through all 12 administrative districts on the island in <1 month. Given that the population of Taiwan has increased >6-fold since then and that the current population is much more mobile and travels more, a future outbreak of a similarly virulent influenza virus strain could conceivably spread through the island in a few days. The total estimated number of excess deaths is 51,048 (95% CI 41,998–61,853). Given the average Taiwan population size during 1918–1920, the percentage of excess deaths was 1.38% (95% CI 1.14–1.68). Given the population of Taiwan at the end of 2007, the number of persons killed by an epidemic of similar magnitude today would be ≈315,000 (95% CI 259,900–384,000). Pandemic readiness planning for Taiwan, or any other country, must consider the potential magnitude of a similar-sized pandemic.

